# Elements of Medicine. Vol. II.—On Morbid Poisons

**Published:** 1842-01

**Authors:** 


### ART. IV.

*Elements of Medicine.* Vol. II.—*On Morbid Poisons.* By ROBERT WILLIAMS, M.D. &c. &c.—*London*, 1841. 8vo, pp. 686.

IN a review of the first volume of this work published in our Tenth Number, we expressed an opinion on the whole favorable to its merits. We acknowledged the ingenuity of the writer, and the extensive information, derived both from reading and experience, which he brought to bear on the subjects of which he treated. At the same time we stated our belief that some of the *laws* which he conceived to have been established were the result of partial induction, and either incompatible with the actual state of knowledge, or likely to prove invalid in its future progress. The present volume is entitled, in full measure, to the praises bestowed on its predecessors, and the legislative part is open to fewer objections because more limited in its scope. The general laws having been laid down in the former volume, those here insisted on relate chiefly to the individual poisons. With respect to these, the author's views will be found, for the most part, consonant with those of the best observers, although his phraseology is peculiar: what he calls the laws of the poison are very often simply what others might call the phenomena and habitudes of the disease; and the only objection to the term *law* is, that it is frequently impossible to reduce such phenomena and habitudes to any fixed law, or determinate sequence. Considered as a series of theoretical and practical essays on disease, the contents of this volume are well worthy of attention: they are evidently the result of much observation and reflection; the author is rich in materials of his own, and where he draws from the resources of others, he has the discernment to adduce only such statements and opinions as are worth listening to. In affirming that much of the peculiarity of this work resides merely in the terms employed, we should not be supposed either to misunderstand the main tendency of the author's reasoning, or to depreciate the importance of the distinctions which he seeks to enforce.

The living body is destined to perform certain functions, which are sustained by means of appropriate relations between its own powers and the agents adapted to call them into operation. In a general way, disease may be said to consist in the failure of such powers, or the disorder of such relations; but there are agents which can, under no circumstances, hold any relation to the living body but one subversive of its healthy functions: such are *malaria*, and the agents, whatever they may be, that produce *plague*, *typhus*, and various other diseases. In contemplating common disease, we witness an increase, diminution, or alteration of the ordinary actions of the system, such as may readily be conceived to arise from impairment of its intrinsic powers, or derangement of its ordinary relations; but in the operation of the noxious agents in question, we see, as it were, an assault upon all physiology, giving rise to a struggle in which the vital force successfully resists an influence repugnant to its laws, or sinks under its malignity. These agents may be called *poisons;* and there is one respect in which they are peculiarly entitled to the name; for whereas the substances usually so called are only relative poisons, the deleterious effect of which is contingent on quantity, and circumstances, *morbific poisons* are absolutely such, and produce disease in all doses, and under all circumstances, in which they can produce a sensible effect of any kind. For example, arsenic, in quantities sufficient to cause a sensible effect, may operate beneficially, or fatally, according to the dose and the circumstances; but marsh miasma, received into the system in quantities sufficient to produce a sensible effect, is uniformly noxious, whatever be the dose or the circumstances. Now it is plain that the morbid phenomena occasioned by the subtile agents under consideration should be placed in a different category from those springing from the ordinary sources of deranged action: their theory must be essentially different; they must demand a different treatment; and we are led to look in different directions for the improvement of practice—expecting it, on the one hand, from an enlarged knowledge of morbid and remedial actions, and on the other, from the discovery of specifics and antidotes. Hence we see reason in Dr. Williams's view of "the propriety of separating this interesting class of disease, singular in its laws and peculiar in its treatment, from the great body of medicine with which it has been so long confounded." (Preface, p. vi.) We cannot, however, go all the way with him in the opinions here expressed. We do not think that enlightened pathologists, in modern times at least, have confounded the diseases in question with the great body of medicine, but that they let them continue attached to it because they did not know how to separate them; and we doubt, also, whether we are even yet in a condition to effect such a separation on any just or permanent grounds. We nevertheless fully admit the general truth of the distinction which our author inculcates, and think that it has been too little kept in view by the majority of medical reasoners: so important indeed do we consider it, that if its distinct enunciation constituted the only merit of the work before us, we should still say that it had not been written in vain. But, in truth, though the poisons and their laws were all left out of the question, enough would remain to render Dr. Williams's book interesting to the medical philosopher and practical physician. Let us proceed, then, to examine some of its particular contents.

The first three subjects, *vaccinia, syphilis,* and *gonorrhœa,* have so recently occupied our attention that we must pass by our author's essays upon them; but we would by no means advise the reader to do so, as they contain much useful information, and many judicious remarks—blended with others which we should be disposed to controvert did space permit. The next subject is *hydrophobia.* Dr. Williams has given a very copious and accurate account of this disease, both in its ordinary forms and occasional varieties; but we must entirely dissent from some of his conclusions. He maintains that the hydrophobic poison may exert its full influence on the nervous system, and rapidly destroy the patient without leaving any visible traces of disease; but that where it has had time to set up all its specific actions, certain morbid changes are always discoverable on dissection, consisting in increased vascularity of the brain, spinal cord, and their membranes, and of the structures supplied by the eighth pair of nerves. He believes that the poison is absorbed, and infects the blood, producing, after a longer or shorter period, functional derangement of the nervous centres and subsequent organic changes in the parts above mentioned. We entirely agree with him that the poison acts essentially on the nervous system; but we differ from him as to the probable manner of its operation. In the first place, it seems to us by no means certain, though we admit it to be most likely, that the virus is absorbed into the blood at all. In traumatic tetanus, or in epilepsy arising from injury of a part distant from the brain and spinal cord, we can no more account for the occurrence of the tetanic seizure, or of the epileptic paroxysm, at one time rather than another after the reception of the injury, than we can account for the access of hydrophobia at a distant and uncertain period from the infliction of the bite; yet in the two former cases there is no virus in the question. That a virus is the cause of hydrophobia there can be no doubt, because the disease is never met with as the consequence of any other wound than that which is poisoned with the saliva of a rabid animal; this virus, however, may be conceived to act, not through its absorption into the blood, but by an impression on the nerves of the part which is propagated to the nervous centres in some manner to us entirely unknown. Secondly, supposing the symptoms to depend on the absorption of the poison, we see no reason to believe that it is taken into the blood immediately after the reception of the bite; on the contrary, we are more inclined to the opinion of those who maintain that the poison is localized in the injured part, and remains inactive till the time when its effects on the system become manifest. No local symptoms of activity are observed till immediately before the eruption of the general disease: then the bitten part usually becomes painful and presents indications of morbid action, the wound sometimes opening anew long after it has been healed, and discharging a sanious matter.

Analogous phenomena occur in traumatic tetanus. In this form of tetanus a morbid state of the nerves of the part, occasioned by a mechanical injury, becomes suddenly influential on the nervous centres by some mode of propagation which we cannot explain: in hydrophobia, one of three things happens: a morbid state of the nerves of the part, originally induced by the action of a poison, is propagated to the nervous centres without any further operation of the poison; or, the poison lying dormant for a time, suddenly begins to be absorbed into the circulation, and thus produces its specific effects; or, lastly, the poison lies dormant for a time, and then, becoming suddenly active, makes a morbid impression on the nerves of the part, which is propagated along their course to the brain and spinal cord, the poison being simultaneously absorbed into the blood, although its absorption be not the essential condition of its activity. The last of these hypotheses appears to us to agree best with the phenomena of the disease, and with what is known of the modus operandi of poisons in general, but we do not wish to be understood as adopting this or any other hypothesis; our only object in these remarks is to show that several opinions are tenable, and therefore none conclusive. Such being the state of the case, it is also uncertain whether the changes observed on dissection are to be regarded as the secondary specific effects of the poison, or merely as the effects of disordered innervation on the vascular actions of the parts. When we consider the similarity of the morbid appearances in hydrophobia and tetanus, and their joint resemblance to those common in most cases of violent and rapidly fatal disease, we are the less inclined to suppose that the changes discovered in bodies which have died of hydrophobia are referrible to the specific action of the poison. We must also remark that our author's notion of their being *secondary* effects, which would of course restrict them to cases of a certain duration, is hardly borne out by a reference to the instances on record, for in some of these the appearances in question have been observed after a disease of very short duration.

There is a question of infinite practical importance which has also a powerful bearing on the pathology of hydrophobia, namely, whether the excision of the bitten part, before the appearance of the symptoms, is or is not certainly prophylactic of the disease? Dr. Williams decides in the negative.

"It may be said that many patients on whom excision or cautery has been performed have escaped hydrophobia; but it is also equally certain that many persons who have submitted to these operations within a very short time after being bitten have, nevertheless, fallen victims to the disease. In a late instance at St. Bartholomew's Hospital, the man had all the bitten parts removed by the knife, and yet died of hydrophobia. A case is also given by Manière, in which actual cautery was promptly used, and to such an extent that the inflammation which followed required a number of leeches to reduce it; yet the patient, though she lived to be pregnant and to miscarry, subsequently died of hydrophobia. Mr. Rogers gives a case in which a patient was bitten by a rabid dog, under such circumstances that cauterization by the argentum nitratum was immediately employed, and repeated in the course of the two next days. The accident happened in July, and the man died in the October following of hydrophobia. A spaniel having been bitten in the ear by a dog suspected of being rabid, Dr. Bent, of Moorshedabad, placed the ear on a board, and with a scalpel cut out a circular piece half an inch all round the wound, and washed the part well with warm water. All this was done in less than a quarter of an hour after the bite was received, which was on the 26th of December, yet on the 11th of January some alteration was observed in the manner and habits of the animal, and on the 14th it died of hydrophobia." (p. 254.)

We must observe, however, that the mass of evidence in favour of the early extirpation of the part is so great that alleged exceptions should be received with extreme caution. Independently of the fallacies arising from imperfect performance of the operation and mistakes in the nature of the case, it should be remembered in all instances where hydrophobia occurs after the destruction of a bitten part, that the virus may possibly have been applied to some slight scratch in another part of the body which has entirely escaped notice. The agitation necessarily attendant on the attack of an animal believed to be rabid, would be very unfavorable to any minute observation or recollection of what might have happened. With respect also to dogs, and other animals covered with hair, it is evident how easily a small wound might escape observation. On the whole, we cannot admit the scanty evidence to the contrary, as subversive of the conviction held by the highest authorities, and based on the most ample experience, that immediate extirpation of the part is an absolute preventive. We think that the experience of Mr. Youatt alone, who, probably, has had more to do with this disease than any other individual, is almost sufficient to decide the point. He has operated in upwards of 400 cases of persons bitten by dogs distinctly rabid, and not one of them has taken the disease.* No one will dispute Mr. Youatt's competency to decide whether a dog be rabid or not; and whichever of the various estimates we adopt of liability to infection after the bite, it is impossible to explain

* Mr. Youatt destroys the part with lunar caustic. We should prefer excision; but the thorough destruction of the part, in whatever way, is the main object.

the immunity of so large a number without a single exception on any other principle than that of the efficacy of the practice. We are much inclined to believe, with Sir A. Cooper, Travers, Hodgson, and others, that the destruction of the part at any time before the actual development of the disease is perfectly effectual. This point, however, not being certain affords a most important subject of enquiry, and every case that can bear upon it should be most diligently recorded. It is no less important in a theoretical than in a practical light, for its decision in the affirmative would involve not only the certain means of safety to the patient, but also an *experimentum crucis* as to the fixation or diffusion of the poison during its latent state.

On the possibility of discovering a prophylactic against the hydrophobic poison, when in its latent state, our author has, we think, been led to a premature conclusion by one of his own general laws. On a former occasion we expressed a doubt of the validity of the law that morbid poisons "are not acted upon by medicinal substances as long as they continue latent." The question, we observed, is "of great interest and importance, although it is one which it must be almost impossible to answer; since the only evidence of a latent period having existed is when that period has terminated."

It must be confessed it is not easy to get hold of anything either in the way of confirmation or refutation; but it just occurs to us that there is one disease arising from a poison which has a sort of bearing on the subject, namely, *ague*. The poison has here the remarkable peculiarity of being alternately active and delitescent: now there is not the least doubt that quinine, given during the intermission (latent state of the poison), will cure the disease. We admit it may be objected that the patient, even during the intermission, is still under the influence of the poison, as evinced by lassitude, &c.; but we merely give this example for want of a better, and as the only one we can hit upon that has any relation to the subject. As far as it goes it makes rather against Dr. Williams's law. At all events such vague generalities should never be allowed to damp our practical researches, and utterly worthless as we believe all known medicines to be in the prevention or cure of hydrophobia, we think it by no means impossible that there may be in nature both a prophylactic and antidote for this horrible disease.

With respect to the origin and propagation of hydrophobia, Dr. Williams states that as far as our knowledge extends, animals of the canine and feline races are alone subject to spontaneous hydrophobia; that in the dog, fox, wolf, and jackall, the disease may arise either spontaneously or from inoculation; that the same is the case with the cat, which is the only feline animal at present known to have this two-fold liability; that the above-named animals, when labouring under hydrophobia, howsoever contracted, generate a poison capable of communicating the disease to man, to every domesticated animal whether bird or beast, and probably to all warm-blooded animals; that those animals in which the disease never originates spontaneously, do not secrete the poison, and that hence, though themselves susceptible of the disease, they cannot propagate it. Though we think it not unlikely that all warm-blooded animals may be susceptible of hydrophobia, we must protest against our author's direct affirmation of the susceptibility of all domesticated birds and beasts. With reference to many of these there is nothing on record to determine whether they are susceptible or not, and we hold it to be of great importance not to take anything for granted on this subject, but to ascertain with scrupulous exactness the relations of the poison in question to the constitutions of as many distinct species of animals as possible, because the conditions of susceptibility may hereafter throw light on the nature and mode of action of the poison. We agree with Dr. Williams in attaching no weight to the unsupported experiment of Magendie and Breschet, in which hydrophobia is alleged to have been communicated by inoculation from a man to a dog. It is much more probable that the dog was previously infected from some other source, than that a result should have been obtained so opposite to those of all former experiments of the like nature. The shadow of doubt, however, thrown by this experiment on the established belief renders it desirable that the question should be set finally at rest by a sufficient number of carefully conducted trials.

The succeeding essay is on the subject of *plague.* Dr. Williams enters at considerable length and with much judgment into the origin of this disease. Clot Bey believes it to originate and to be endemic along the whole eastern and southern coast of the Mediterranean, the principal centres being Egypt, Syria, and Constantinople; the intensity of the disease diminishing as we approach Greece and the Adriatic on the one side, and Barbary and the Straits of Gibraltar on the other. Dr. Williams observes that further evidence would be necessary to prove so extended an origin; but that all authors are agreed that Egypt does give origin to the plague, and most of them consider it as the great and primary, if not the sole source, of the morbific miasm. There seems also reason to believe that the generation of the poison is limited to certain very circumscribed portions of Egypt. This is the opinion of Clot Bey, who observes that the plague is more rare in Upper than in Central Egypt, and in Central than in Lower Egypt, and more frequent on the coast of Syria and the shores of the Bosphorus than in the interior of Asiatic or European Turkey. Admitting the origin of plague in certain limited districts of Egypt, two hypotheses, says our author, have been advanced to account for the poison which produces it. The one is that it is engendered among the lower classes of the people, who live in a state of extreme filth and misery, and produces a disease propagated solely by contagion; the other is that the poison, however generated, exists in the atmosphere, and produces a highly contagious disease, or a disease in which the body of the patient evolves a poison which is both infectious and contagious. After enumerating the various sources to which the origin of the plague has been referred, Dr. Williams concludes:

"It is probably in vain further to attempt to penetrate the obscurity in which the remote causes of this disease are involved, or to assign a sufficient reason why this disease, unknown, as it is conceived, in the days of the Pharoahs and of the Ptolemies, has continued to rage for the last 1300 years with unabated violence in Egypt and perhaps in the neighbouring countries. It seems certain that the geological structure of Egypt, the overflowing of its river, and the conditions of its atmosphere have undergone no change. It must be admitted, however, that the husbandry of the Egyptians has undergone a most remarkable alteration; the cultivation of rice having succeeded to that of corn, and so also have their manners and habits. The squalid misery and mud-built towns of the present day, forming a strong contrast with the many magnificent and stupendous monuments which remain to attest the former luxury, opulence, and splendour of the cities of ancient Egypt. The poison of the plague, therefore, like that of the smallpox, the scarlet fever, or the measles, seems to be engendered by the action of physical causes as yet unappreciable. But they must be permanent in Lower Egypt, for sporadic cases prevail all the year round in that district. The space in which they are extricated, however, it has been shown must be limited to narrow bounds, for this disease is rare in Upper Egypt, and has never been known to pass the first cataracts. The hospitals, likewise, which were formed in 1834-5, along the borders of the desert, and many regiments encamped in that position almost entirely escaped the attacks of the plague which raged on the banks of the Nile. It was remarked also that several villages suffered little loss in that epidemic, although in the immediate neighbourhood of Cairo, where the disease was terrifically fatal." (pp. 267-8.)

There is nothing so peculiar in Dr. Williams's opinions respecting plague as to demand any special notice, and we must therefore content ourselves with recommending his article as a very full and able review of what is known on the subject. We may at the same time deduce from it a justification of our remark that Dr. Williams sometimes bestows the name of *laws* on certain facts and phenomena sufficiently well known under less emphatic denominations. It is admitted that the plague is produced by a poison; what then are its laws? The passage just quoted sufficiently shows that we know not the manner, nor even the circumstances in which the poison is generated. Are we any better acquainted with the causes which influence its activity, and convert the endemic disease of certain districts into a wide-spreading pestilence? Is the mode of its propagation from one individual to another distinctly ascertained, or is it absolutely certain that the disease is contagious at all? Do the symptoms succeed each other in any order which can be considered as at all determined? Are the characters of the eruption or the textures in which it appears in any degree uniform? Or does the eruption, in whatever form it may present itself, observe any definite duration or hold any distinct relation to the symptoms? All these questions, we fear, must be answered in the negative, and, if so, it is evident that we know nothing of the laws of the poison, and that our author in attempting to unfold them has merely given us a history of the disease.

We have now to consider *cellulitis venenata*. By this term Dr. Williams designates the disease which results from punctures received in dissection or any other inoculation with a certain virus existing in the dead body. He observes that there is great difficulty in determining whether the poison producing this disease be uniformly the same and a simple product of animal decomposition, or whether it be a product of the disease of which the patient has died, and variable according to the nature of that disease. He concludes, however, that there is not a variety of poisons but one peculiar and specific poison. This he conceives to be proved by the uniformity of the phenomena resulting from punctures received in the examination of bodies which have died of the most opposite diseases, and of bodies which have not died of any disease at all, as those of slain animals. Dr. Williams notices in illustration the case recorded by Dr. Spurgin, of a cook-maid who received it from the body of a stale hare, and another in which the injury was received in hanging up a piece of fresh meat. He then cites a case given by M. Morand, (Hist. Acad. Royale des Sciences, 1766, p. 54.)

"A large ox, unable to travel (mal à butin), was killed and cut up by a butcher, who put his knife for a short time into his mouth, and was soon after seized with swelling of the tongue and throat, and also with universal gangrene, so that he died on the fourth day. An innkeeper pricked his hand with a bone of the same ox, and a livid tumour formed on the part, the arm sphacelated, and he died on the seventh day. Some drops of the blood of this animal, also, fell on the hand of his wife, and the hand swelled and a tumour formed, which she had great difficulty in getting cured." (p. 334.)

Another example is adduced from Rust's Magazine in which ten individuals died of anthrax, in the district of Merseberg, in the year 1822, and it was ascertained that several of them had been employed in skinning sheep which had died of *blutseuche.** The cases here cited from the French and German works are, we think, not at all to the purpose: there is no mention of any puncture or abrasion which, generally speaking, seems essential to the production of the disease under consideration, and the cases are probably referrible to the head of those obscure animal poisons accounts of which are scattered through medical writings, and which are evidently distinct from that generated by simple animal decomposition. Dr. Williams coincides in the opinion, fully justified by the experience of anatomists, that a body recently dead is much more dangerous than one in an advanced stage of putrefaction. We may observe, in passing, that this is worth keeping in view, in relation to the question lately so much agitated, of the influence of animal putrefaction on health; for it suggests that such influence may be very various according to the different stages and circumstances of the process, and that the diversity may extend to the exhalations from the body, as well as the fluids contained within it.

Dr. Williams's view of the pathology of the affection seems to be fairly deducible from an attentive consideration of its phenomena.

"The theory of this disease is, that a poison, whatever be its intimate nature, is absorbed and carried into the circulating torrent, and after a short period of latency, usually occasions only a diffuse inflammation of the wounded part, without any serious affection of the constitution. In other cases, however, in addition to the diffuse inflammation of the part or limb, a severe form of fever is added with extreme prostration, coated tongue, and disturbed alimentary functions, and as the disease proceeds, abscesses are often formed in various parts of the body, remote from the original wound; usually beneath the great pectoral muscle, although not in the course of the absorbents leading from the hand to the subclavian vein. The poison therefore acts locally on the punctured limb, on the cellular tissue generally, and also on the great nervous centres, producing the phenomena of fever. The local phenomena, when the poison is of sufficient intensity to produce disease of any moment, are inflammation of the punctured part, involving not only the hand, but also the arm, and oftentimes extending as far as the axilla. A more intense form of the disease, however, is when the inflammation of the punctured part is slight; but severe inflammation commences in the axilla, extending over the neck and down the side, and sometimes attacking remote and even opposite parts of the body. This inflammation may terminate by effusion of serum, by suppuration, or by gangrene." (pp. 337-8.)

* *Blood-pestilence;* called by English veterinarians *the blood,* or *blood-striking.* It is analogous to the *quarter-evil* of young cattle.

The phenomena of cellulitis venenata are illustrated by a variety of cases, none of which, however, are derived from the author's own experience. His remarks on the controverted treatment of this affection leave us in the dark as to that which he would himself prefer. He observes, with truth, that slight cases generally do well, while in those of a severe kind which have been recorded, one half of the patients have recovered and the other half have died. Under these circumstances he does well, we think, to suspend his opinion, which we also beg leave to do, intimating at the same time a suspicion that a more decided use of stimulants and opiates from the commencement might have succeeded in some cases where they failed from being used only after antiphlogistic treatment had been tried ineffectually.

Dr. Williams next treats of the "poison of farcinoma," and gives, under this head, an excellent review of what is hitherto known concerning glanders, whether in the equine animals or in man. The subject not demanding any particular notice from us at present we pass to that of "the poison of porrigo." The author's delineation of this disease is very good; but we cannot yield an unlimited assent to his pathology.

"The theory of this disease is, that the poison is absorbed and infects the blood, and after a given period of latency produces a pustular eruption of a given character on the part of the scalp to which it is applied, and subsequently, perhaps, on the whole scalp. A similar eruption sometimes appears on other parts of the body to which the poison may have been immediately applied, but not otherwise. The proof of the blood being infected in this disease is, that there are cases in which the head has been shaved and carefully washed for many months, and each favus destroyed by lunar caustic as soon as it has appeared, yet the whole scalp has ultimately been covered by them, and, as far as could be judged, without any direct application of the poison." (pp. 402-3.)

This reasoning is surely very inconclusive. In the first place our author takes for granted, what has never been proved, that porrigo may not be generated in the individual, independently of all contagion; and, secondly, supposing the disease to be always the result of an extraneous poison, may not this poison be local in its operation? and may not a morbid action caused by a poison be propagated by the sympathy of contiguity like any other diseased action? It appears to us that our author has been led, by the too implicit adoption of an exclusive theory of the operation of poison—that of their absorption into the blood—to lose sight of one of the most interesting questions connected with these agents, namely, their *localization.*

The diversities of medical experience are truly curious. It is notorious that scald-head is a disease from which the doctors in general derive very little credit, and which is considered particularly intractable. Dr. Williams however states that during the twenty years he has been attached to St. Thomas's Hospital he does not remember a single case in which the treatment adopted has failed. We believe we may say that we have been equally successful in all the cases which have occurred to us for some years past. The following is Dr. Williams's account of the treatment which has been attended with such happy results:

"The London Hospitals do not, in general, admit cases of porrigo into their wards, yet in the course of a year, the number received is, notwithstanding, considerable; and the practice adopted by Drs. Roberts, Powell, and Haworth, while I was a student at St. Bartholomew's Hospital, in every case of porrigo, whatever the stage or form of the disease, was as follows:

"They first directed the head to be poulticed till all the scabs were removed, and this being effected, they shaved the head, and applied the unguentum picis liquidæ over the whole scalp, which they directed should be washed off night and morning with soft soap and water, and the ointment to be repeated. The patient was also to wear an oil-skin cap, and his head to be shaved twice a week. This treatment was so universally successful in their hands, that I have adopted it during the twenty years I have been attached to St. Thomas's Hospital, and I do not remember one instance in which it failed. Indeed, the only improvement I have been able to make on it has been in cases in which the favi are still unbroken, or the incrustation but trifling, to wash the whole scalp night and morning with the oleum terebinthinæ purificatum, by means of a common shaving brush, taking care to prevent its running into the eyes." (pp. 411-12.)

For our own part, it was repeated observation of the failure of all set plans of treatment that induced us to treat porrigo as we now do with uniform success, on the ordinary principles of medicine; that is, by applying sedatives when the parts are irritable, and stimulants when they are indolent; trusting to topical means when the disease appears to be dependent upon local causes, and exhibiting constitutional remedies where the general health appears to be deranged. The last-mentioned consideration is too little attended to, and we can affirm from experience, that where porrigo occurs in weakly and cachectic children, alteratives and tonics will go further to cure it than any local means whatever. We have found sarsaparilla particularly useful in these cases.—The doctors in general, then, fail continually in curing porrigo; Dr. Williams always cures it by observing a plan; and we always cure by eschewing all plans.

The succeeding essay is on "the paludal poison." It is by far the longest in the book, and seems to us to be of very unequal merit. It is divided into two portions, the first treating of paludal fevers, and the second of dysentery.

We consider our author's remarks on the generation of the marsh poison so ingenious that it was our intention to have given a recapitulation of his general conclusions respecting it. We find, however, that our space will not admit of it, and we must therefore be content with recommending this portion of the work as a good example of the manner in which such enquiries should be conducted.

The various forms of marsh fever are well illustrated; the author, however, has made no mention of that highly congestive form which has obtained the name of febris algida, *fièvre algide.* The strong analogy of this to malignant cholera renders it an object of particular interest. It is, we believe, the only disease except cholera in which the pulse is frequently extinct for many hours before death, and the temperature after death sometimes exceeds that of the body while alive.

In the article *dysentery*, Dr. Williams adopts two fundamental principles which are so obviously erroneous that it would be futile to follow up his reasoning. These are, first, that the paludal poison is always the remote cause of dysentery; secondly, that to produce dysentery the poison must be applied in a dose less than that which produces the mildest form of paludal fever. That dysentery is a very frequent disease in countries infested with malaria no one will dispute, but that malaria is the sole cause cannot for a moment be admitted. It is in the face of all observation, and it is curious enough that Dr. Williams should furnish us with an excellent confutation of his own doctrine. Speaking of the influence of diet, he says:

"At St. Helena, more than one third of the admissions and nearly two thirds of all the deaths among the troops have been from diseases of the stomach and bowels; *of these dysentery is the prevailing form, and is even more severe than in the West Indies; yet in vain do we seek for any peculiarity in the climate or locality to account for it.* In consequence of the repeated representations to which the frequency of this disease gave rise, a board of medical officers was, in October, 1836, directed to investigate the subject, who came to the conclusion that the health of the troops had been manifestly impaired by the constant use of salt rations. Two days' fresh provisions per week were in consequence ordered for the troops, with the privilege of exchanging a portion of their salt meat for fish or vegetables. The beneficial effects of this alteration was shown by the cases of visceral obstructions being reduced to half their previous amount in the course of the following year, *and now they are said to be comparatively rare.*" (pp. 546-7.)

The grounds on which our author adopts his notion of the relative doses of paludal poison necessary to produce dysentery and fever are so truly singular that we regret that we cannot afford space to transcribe them. But they are utterly invalid.

The next subject is *Cholera Indica*. Dr. Williams has given a very good essay on the subject, keeping generally close to the facts, and reasoning on them with ability. With respect to the theory of the disease he falls into his favorite train of thought, and concludes that the morbid phenomena are the result of a poison absorbed into the blood, on which all we have to say is that it may be so for anything we know to the contrary.

The only remaining subject is "the poison of influenza." Dr. Williams's observations on this disease will very well repay the trouble of perusal, though they are imbued with the theoretical spirit which is too prominent throughout the work. He seems less certain about the laws of this poison than those of some others; and as this article has extended to a considerable length we will waive a subject on which our remarks might not perhaps tend greatly to edification.

Before dismissing this work it is our unpleasant duty to animadvert on the defective style in which it is written, and the manifest carelessness with which it has been allowed to pass the press. Some slight instances will be found in the few passages we have extracted; and one cannot read many consecutive pages of Dr. Williams's book without encountering some clumsiness of diction, if not some absolute solecism in grammar. We notice a few of these, opening the volume almost at random, to show that we are not unjust or captious in our remarks. At p. 251 is the following sentence: "The patient at the time of the operation is represented as being furiously insane, and his mind so overthrown as to require to be confined in a straight waistcoat," i. e. the patient's *mind* required to be confined in a straight (strait-) waistcoat. At p. 216 we meet with the novel term *parotiditis*. At p. 232 we read "the dog's tooth is indeed "*a fomites*." At p. 360 we find that "Dr. Osborne has proposed pledgets dipped in the *Træ cantharides;* and we may observe that the word tincture is continually put in the genitive where it ought not to be. At p. 365 we meet with *veterinists* instead of *veterinarians;* and throughout the work we find the poisons capable of coexisting with others designated by the term *Coexists*—certainly one of the most extraordinary substantives ever hatched. Then we find twice within the space of half a page the words *bubo d'emblée* instead of bubon d'emblée, *principle* for principal, *suffosis* for suffossis, and such like. We must observe, also, that several quotations are stultified by misprints or wrong references.

All these blemishes, and many more of the like description, we hope to see removed in the next edition of the book, which we shall be very happy to see it attain. Taking its excellencies and its faults together, and we have endeavoured to indicate both according to the best of our judgment, we have no hesitation in recommending Dr. Williams's work to all who are interested in the philosophy or practice of medicine.

---

